# In Vitro Hypoglycemic and Anti-Inflammatory Potential and Toxicity of Powders from Pulp and by-Products of *Ziziphus mistol* from Argentina

**DOI:** 10.3390/foods11142125

**Published:** 2022-07-18

**Authors:** María Eugenia Orqueda, Sebastian Torres, Iris Catiana Zampini, María Inés Isla

**Affiliations:** 1 Natural Products Research Laboratory (LIPRON), Institute of Bioprospecting and Plant Physiology (INBIOFIV-CONICET-UNT), San Lorenzo 1469, San Miguel de Tucumán 4000, Argentina; eorqueda@yahoo.com.ar (M.E.O.); sebatk@hotmail.com (S.T.); zampini@csnat.unt.edu.ar (I.C.Z.); 2 Biolates Network for Sustainable Use of Ibero-American Vegetable Biomass in Cosmetics (Biolates CYTED), San Miguel de Tucumán 4000, Argentina; 3 Facultad de Ciencias Naturales e IML, Universidad Nacional de Tucumán, San Miguel de Tucumán 4000, Argentina

**Keywords:** *Ziziphus mistol*, hypoglycemic effect, anti-inflammatory effect, pulp powders, by-products powders

## Abstract

Background: The *Ziziphus mistol* fruit (vulgar name mistol) is used in northwestern Argentina in traditional food and beverage preparations and popular medicines for liver and respiratory disorders. Aims: The aim of this research was to evaluate the hypoglycemic and anti-inflammatory activity in pulp powders and sub-products (skin and seeds) of mistol fruit, along with their toxicity. Methods: Powders from mistol seeds, pulp, and skin were obtained. Antioxidant capacity and inhibitory activity against key enzymes involved in metabolic syndrome were determined by in vitro assays. Results: The mistol powders obtained from the different fruit parts reduced glucose bioaccessibility. Before and after simulated gastroduodenal digestion, the polyphenol-enriched extracts (PEE) obtained from mistol powders increased glucose uptake by yeast cells and inhibited the pivotal enzymes of the inflammatory pathway (cyclooxygenase-2, lipooxygenase-1, and phospholipase A_2_). The analyzed mistol powders did not show acute toxicity or genotoxicity in model organisms and cell cultures. Conclusions: These results evince the potentiality of both the pulp from *Z. mistol* fruits and residual biomass (seeds and skin) to obtain biofunctional powders to use as supplements for metabolic disorders associated with chronic diseases.

## 1. Introduction

**Figure 1:**
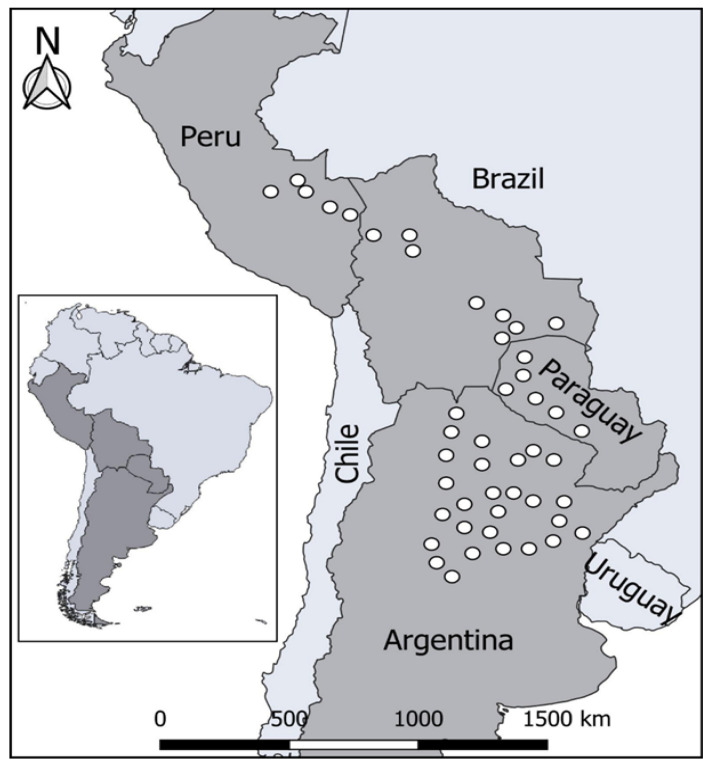
Mistol distribution map in South America.

Chaco forest in Argentina is undergoing a significant process of degradation of its biodiversity due to deforestation, desertification, and changes in the land use [1]. The genus *Ziziphus* includes approximately 170 pantropical species, 25 of which are native to the Americas and the Caribbean [2]. The genus is represented by trees and shrubs that can be found mainly in arid environments, due to their ability to adapt to water shortage. Various studies on the reproductive biology of the genus have shown a broad diversity in the reproductive system of *Ziziphus* [3]. *Ziziphus mistol* Griseb (Rhamnaceae), commonly known as mistol, is an edible tree that grows in the Chaco forest; together with *Aspidosperma quebracho-blanco*, *Cercidium praecox* Burkart and Carter, *Geoffroea decorticans* Burk, and *Prosopis nigra* (Griseb) Hieron [4], it constitutes the essential matrix corresponding to the semi-arid Chaco woodland. It is a thorny deciduous honey plant (10–15 m tall) widely spread throughout Perú, Bolivia, Paraguay, and Argentina (Figure 1). It has a smooth bark and its branches are pubescent, zigzagging with long, sharp spines located in pairs at the nodes. It is a semi-evergreen tree with simple, deciduous leather-looking leaves and margins that are barely toothed and parallel to each other. The fruit is a globose drupe approximately 1.5 cm in diameter with a pasty and sweet pulp [5]. Although the *Z. mistol* tree is an endangered species, it is classified as a species with “insufficient data” (DD) according to the IUCN [6,7] red list. That is a consequence of the anthropogenic action throughout the last decades that has degraded Chaco forest natural resources at the expense of promoting the advancement of soybean crops and the extension of the agricultural border. Although the mistol is not extensively cultivated, it provides a wide variety of non-wood forest products derived from fruits, bark, and leaves. The fruits can be used as food in the manufacture of typical foods and drinks in northwest Argentina, and in traditional medicines for liver and respiratory disorders. The fruits and leaves are used as expectorants, disinfectants, skin healings, and antidotes for poisonous insect bites. The bark is astringent, contains saponins, and is used by locals to wash clothes and used to feed domestic animals [8]. The fruit is a globose drupe, reddish-brown, approximately 1.5 cm in diameter with a sweet, pasty pulp [8]. Mistol powders were previously studied for their nutritional and phytochemical composition and functional properties [8]. According to this study, the different mistol fruit powders have low sodium and lipid contents, as well as high contents of proteins, potassium, and fibers. Additionally, all of them, particularly the seed powder, have a high level of flavonoid compounds. Chalcones and derivatives of quercetin, apigenin, and kaempferol are highlighted in the chromatographic profiles [8]. The polyphenolic-enriched extracts obtained from mistol seed, pulp, and skin powders have an antioxidant capacity and inhibitory activity on key enzymes, such as α-glucosidase, α-amylase, and lipase [8], involved in the metabolic syndrome.

Recent studies have shown that there is a relationship between inflammation and metabolic abnormalities in diabetes, which leads to endothelial injury and the development of vascular complications [9]. When produced chronically in diabetic patients, the inflammatory mediators produced by the arachidonic acid metabolic pathway trigger pathological processes that culminate in major complications. A recent analysis showed an association between higher levels of inflammatory markers and the incidence of type II diabetes mellitus [10]. For this reason, the search for natural compounds that inhibit the enzymes responsible for the production of these mediators is proposed as a key therapeutic objective to prevent or improve renal and cardiovascular complications in diabetes [11]. Considering this background and the functional traits of *Z. mistol* fruits, the present work aimed to evaluate the hypoglycemic activity and inhibitory activity of key enzymes in the inflammatory pathway by polyphenols from seed, pulp, and skin powders of *Z. mistol.*

## 2. Materials and Methods

### 2.1. Plant Material and Powders

Ripe fruits of *Z. mistol* Griseb were collected from different wild trees from February to March 2015 in Fernández, Santiago del Estero, Argentina (27°55′25.1″ S; 63°53′7.0″ W). The plant material was identified by Dr. Soledad Cuello, a botanical taxonomy expert, and entered into the herbarium. The correct name of the selected species was checked in “The World Flora Online” [12]. Seeds, pulp, and skin were obtained from the fruits, lyophilized, and crushed to powders, which were then vacuum packed and kept at −20 °C.

### 2.2. Polyphenols-Enriched Extracts

The polyphenols-enriched extracts (PEE) were obtained from pulp, seed, and skin powders of *Z. mistol* fruits. The extract production (1 g powder in 5 mL 95° ethanol) was carried out at 25 °C in three successive extractions until exhaustion of all phenolic compounds. Each extraction was assisted by ultrasound for 30 min. Then, the samples were put together, filtered, and dried in a vacuum stove.

Total phenolic content in the PEE was determined by Folin–Ciocalteau assay [13], and the results were expressed as mg of gallic acid equivalents (GAE)/100 g of powder of each fruit part.

### 2.3. Simulated Gastroduodenal Digestion of Mistol Powder and PEE

The mistol skin, pulp, and seed powders (0.5 g), as well as the PEE, were subjected to a simulated gastroduodenal digestion process (GD) following the methodology described by Orqueda et al. [8]. Powder suspensions were centrifuged at 10,000× *g*, and both the digested and non-digested powders were used to determine the adsorption and diffusion capacity of sugars. After the GD process, the phenolic compounds were recovered with ethyl acetate. The recovered organic phase was dried and resuspended in DMSO (1 mg GAE/mL) for subsequent analyses.

### 2.4. Hypoglycemic Activity

#### 2.4.1. Glucose Adsorption of Mistol Powders

The glucose adsorption capacity of each powder sample obtained from mistol both before and after GD was analyzed according to the methodology proposed by Orqueda et al. [14]. For the test, 10 mL of glucose solution (20 mM) and 0.5 g of powder from each part of the fruit were mixed and incubated at 37 °C for 4 h, agitating frequently. Then, the mixtures were centrifuged (4000× *g* for 20 min), and the free glucose content was determined in the supernatants. Glucose bound to the mistol powders was calculated:

Glucose bound: [(G1 − G2)/(Weight of the sample)] × volume of solution

G1: glucose concentration in the initial solution (20 mM).

G2: free glucose concentration after 4 h.

#### 2.4.2. Mistol Powders’ Effect on Glucose Diffusion

The impact of the mistol powders on in vitro glucose diffusion was determined according to a previous method [14]. Briefly, the mistol powders, both before and after GD, (0.25, 0.5, and 1 g) were put in contact with 5 mL of glucose solution (20 mM) and dialyzed against 40 mL distilled water at 37 °C. The glucose content in the dialysate was determined by using the enzymatic glycemia kit (Wiener lab. 1400101, Rosario, Argentina). A negative control without mistol powder samples was performed.

#### 2.4.3. Improvement in Glucose Uptake in *Saccharomyces cerevisiae* Cells by Mistol PEE

The analysis was carried out according to a previous protocol [14]. A yeast suspension (100 µL) in distilled water (10%; *w*/*v*) was mixed with each PEE before and after the simulated GD process (10–100 mg/mL). Then, 1 mL of 20 mM glucose solution was added to this mixture, incubated for 1 h at 37 °C, and centrifuged at 2500× *g* for 5 min. The glucose concentration in the supernatant was determined by using an enzymatic glycemia kit. The percentage increase in glucose uptake by *S. cerevisiae* cells was calculated.

### 2.5. Anti-Inflammatory Activity of Mistol PEE

In all cases, the percentage of inhibition of each inflammatory enzyme was calculated at a fixed concentration of 100 μg GAE/mL. Commercial anti-inflammatory drugs were used as positive controls for the test.

#### 2.5.1. Inhibition of the Enzyme Phospholipase A_2_ (sPLA_2_)

The activity of the sPLA_2_ enzyme was analyzed according to the method described by D’Almeida et al. [15] using 1,2-diheptanoylthio-glycerophosphocholine (1,2 dHGPC) as the substrate. The reaction mixture contained 50 µL of Tris-HCl buffer (10 mM, pH 8), 10 µL of 10 mM 5.5’-dithiobis-2-nitrobenzoic acid (DTNB), 10 µL of sPLA_2_ enzyme (1 µg/mL), and 100 µg GAE/mL of the tested samples dissolved in DMSO. The reaction was started by adding 150 µL of 1.66 mM 1,2 dHGPC and held for 20 min at 25 °C. Absorbance was measured on a microplate reader (Biotek ELx808, Gen5™ Data Analysis Software, Agilent, Santa Clara, CA, USA) at 414 nm every 2 min for 20 min. The commercial anti-inflammatory drug Naproxen was used as the reference compound (50 μg/mL).

#### 2.5.2. Inhibition of the Lipooxygenase Enzyme (LOX)

The test was carried out following the methodology proposed by D’Almeida et al. [15]. The reaction mixture contained enzyme (0.9 mM soybean LOX-1), substrate (50 μM linoleic acid dissolved in 0.2 M sodium borate buffer pH 9), and the PEE. The absorbance of the reaction was measured every 30 sec for 5 min at 234 nm. Indomethacin was used as the reference compound (50 μg/mL).

#### 2.5.3. Inhibition of the Cyclooxygenase Enzyme (COX)

The inhibitory activity against the COX-2 enzyme of the mistol seeds, pulp, and skin extracts was measured by using a COX inhibitor detection assay kit (Cayman Chemical, Ann Arbor, MI, USA) through the production of prostaglandin (PG) by ELISA. PG from arachidonic acid was obtained through the action of the recombinant human COX-2 enzyme. Inhibitory tests were carried out with different concentrations of mistol extracts (5–100 μg GAE/mL) or nimesulide (0.25–2.0 µM). The percentage of inhibition of PGE_2_ production was calculated for each extract of the different parts of the fruit.

### 2.6. Toxicity Assays

#### 2.6.1. Acute Toxicity

The toxicity test using *A. salina* has been used for decades as a rapid detection method on a laboratory scale. This technique has proven to be highly advantageous due to its simplicity and low cost, its good correlation with other animal testing methods, and the possibility of evaluating a large number of samples at the same time and in a short period of time. Increasing concentrations of each PEE (2.5–1000 μg GAE/mL) were used to determine its acute toxic effect by utilizing the *A. salina* microplate assay [16]. Negative controls with DMSO and positive controls with potassium dichromate (10–40 μg/mL) were assayed. After 24 h of exposition, the number of dead shrimps in each well was counted.

#### 2.6.2. Salmonella Mutagenicity Assay

*Salmonella Typhimurium* strains TA98 and TA100 were used to evaluate the possible mutagenic effect of PEE (125 to 500 µg GAE/plate) [17]. The 4-nitro-o-phenylenediamine reagent (4-NPD, 10 µg/plate) was used as a positive control. Three plates per dose and two separate sets of experiments were performed in each case. The results were expressed as the number of revertants/plate, and the Mutagenicity Ratio (MR) was also calculated, which is the ratio between the number of test plate revertants (induced revertant, IR) and the number of revertants on the control plate (spontaneous revertant, ER): MR = IR/ER. The samples were considered mutagenic when the revertant average number was twice as much or higher than the spontaneous revertants or if the MR was above two [17].

#### 2.6.3. Cytotoxicity Tests

GM07492-A (human fibroblasts) cell lines were kindly provided by Laboratory Mutagenesis, University of Franca, São Paulo, Brazil. The cell line was cultured in HAM-F10/DMEM medium (1:1) supplemented with 10% Fetal Bovine Serum (FBS) and containing 1% penicillin/ampicillin at 36.5 °C in a humidified 5% CO_2_ atmosphere. For the experiments, 1 × 10^4^ cells were seeded on 96-well plates containing 100 µL HAM-F10/DMEM medium supplemented with 10% FBS and were incubated at 36.5 °C for 24 h. After this period, the treatments were carried out for 24 h using each extract at concentrations between 0 and 1000 µg/mL. Cell controls, i.e., no treatment, and solvent (1% DMSO) controls were included. Cell viability was determined with the MTT tetrazolium salt assay [17]. The results are expressed as IC_50_ (concentration in µg/mL that inhibits 50% of cell growth).

### 2.7. Statistical Analysis

The values were determined at least three times with three individual samples. Each experimental value obtained was expressed as the mean ± standard error (SEM). A one-way ANOVA with a Tukey post-hoc test with a confidence level of 95% was used for group comparisons. The analyses were carried out by using the statistical software InfoStat (2015, Grupo InfoStat, Universidad Nacional de Córdoba, Córdoba, Argentina) and R studio software (2022.02.3, RStudio.Inc., Boston, MA, USA).

## 3. Results and Discussion

### 3.1. Hypoglycemic Effect of Mistol Extracts and Powders

The malfunction of the regulatory mechanisms of the glucose metabolism is a primary hallmark of metabolic disease. Complementary natural strategies are needed to prevent or improve this malfunction and prevent the progression of normoglycemia to prediabetes and type-2 diabetes throughout the life of patients at risk [18]. Mistol fruits are excellent sources of natural functional food ingredients conferring health benefits. The mistol pulp, skin, and seed extracts were previously characterized by Orqueda et al. [8], and numerous chemical compounds were detected, including flavonoids and procyanidins, many of which featured biological activities. Among these, derivatives of quercetin, rhamnetin, and kaempferol were detected. Previous studies have demonstrated that these compounds show antioxidant capacity and inhibitory activity of enzymes related to carbohydrate metabolism [8]. For this reason, the ability of mistol powders to adsorb glucose or to promote glucose diffusion, as well as the effect of mistol PEE on glucose uptake by cells, was evaluated. These assays are often used to study the effects of plant extracts and natural compounds on glucose bioavailability and glucose absorption and, therefore, their antihyperglycemic and antidiabetic potentials [19,20].

#### 3.1.1. Glucose Adsorption Capacity of Mistol Powders

**Figure 2:**
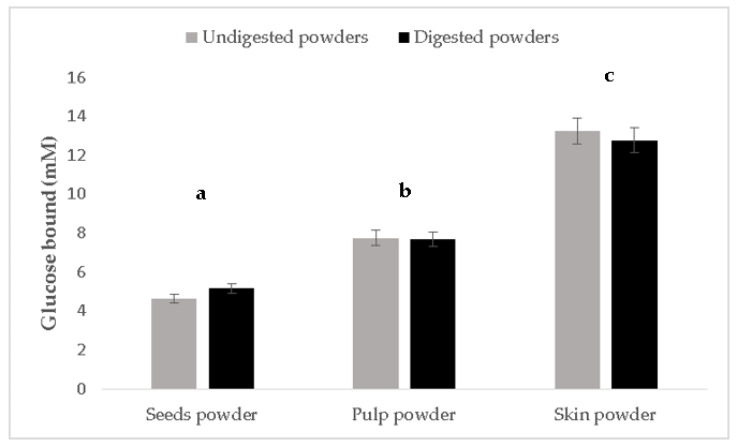
Glucose adsorption capacity (determined by measuring the mM of glucose in dialysate) of digested and undigested mistol powders. Values are presented as mean ± SEM and scrutinized by 1 or 2-way ANOVA, followed by a Tukey or LSD test using R studio software. Different letters on the bars indicate significant differences between the three parts of the fruit, evaluated according to Tukey’s test (*p* ≤ 0.05). No significant differences (*p* ≤ 0.05) were observed between digested and undigested powders.

Figure 2 depicts the ability of both digested and undigested mistol powders to bind glucose at 20 mM concentration. All mistol powders effectively bound the glucose, and no significant differences (*p* > 0.05) were observed between undigested and digested samples. The mistol skin powder was the most active in the adsorption of glucose (about 65% adsorption), followed by the pulp, and then by the seed powder (Figure 2). This property is probably due to the higher amount of fibers present in mistol skin, in comparison to the other parts of the fruit, widely reported as hypoglycemic [21]. Fibers often provide a diffusion barrier due to their high viscosity and ability to bind glucose [22]. The skin powder has the highest amount of crude fiber (23.2%) compared to the other fruit parts, namely, 13.8 and 18.8% for the skin and pulp powder, respectively [8]. However, besides the quantity of fiber, the chemical composition can influence the viscosity of the gels [23]. Additionally, dietary fibers can significantly reduce the transit time in the gastrointestinal tract of ingested food, a fact which can be translated as less time available for di- and polysaccharides in the diet to be digested and absorbed [22].

#### 3.1.2. Effect of Mistol Powders on Glucose Diffusion

**Figure 3:**
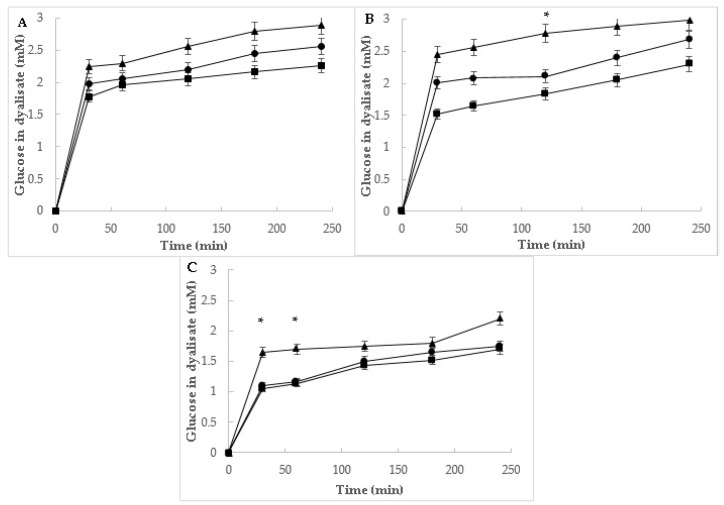
Effect of different amounts (0.25 g, ▲; 0.5 g, ●; and 1.0 g, ■) of mistol powders on glucose diffusion: (**A**) seed, (**B**) pulp, and (**B**,**C**) skin powders. Results are expressed as the mean of three independent tests with SEM. The symbol * at a given time indicates significant differences (*p* ≤ 0.05) between the glucose content in the dialysate between the three amounts of mistol powder, according to Tukey’s test.

The ability of mistol powders to delay glucose diffusion through a dialysis membrane was evaluated, simulating passage through the intestinal mucosa. The most active portion of the fruit was again the skin, allowing only 15% of the glucose tested to diffuse through the dialysis membrane (Figure 3). Once more, no significant differences were observed between the digested and undigested powders. These effects could also be attributed to the dietary fibers that could form highly viscous gels that entrapped glucose. Similar results showing the delay of glucose diffusion through membranes by fruit and plant fibers were previously reported [21]. These beneficial effects on glucose metabolism suggest specific strategies and biological contexts that can be exploited to maximize the antidiabetic benefits of mistol powders; however, additional in vivo research is also warranted.

#### 3.1.3. Effect of Mistol Powders on Glucose Uptake by Yeast Cells

**Figure 4:**
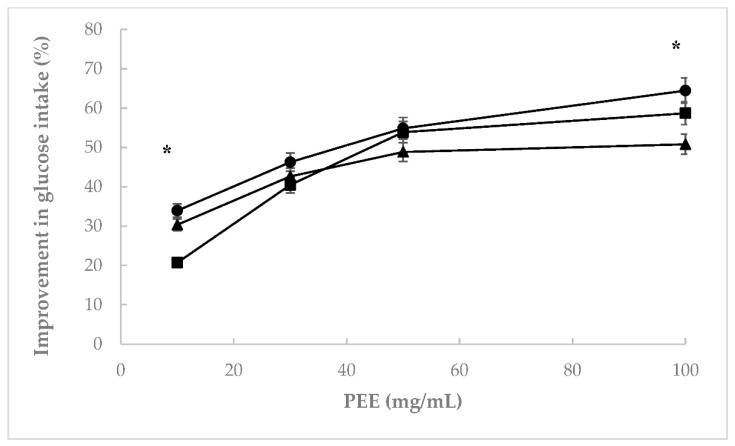
Effect of mistol extracts (PEE) on glucose consumption by *S. cerevisiae*: Skin (■), pulp (●), and seed (▲) PEE. Results are expressed as the mean of three independent tests with SEM. The symbol * at a given extract concentration indicates significant differences (*p* ≤ 0.05) between percentage of improvement in glucose intake by yeast cells between the three parts of mistol fruits, according to Tukey’s test.

All *Z. mistol* extracts efficiently improved the transport of glucose through the cell membranes of yeast cells (Figure 4). A positive dose–response effect was observed for all samples. The seed samples were the most effective at the highest concentration tested (100 mg/mL). The study of glucose transport across the yeast cell membrane has been considered a meaningful in vitro technique that is effective for the screening of natural compounds with hypoglycemic activity [24]. Since glucose transport across the yeast cell membrane occurs via facilitated diffusion and is promoted by specific membrane carriers, i.e., those that transport glucose down a concentration gradient, an increase in the glucose uptake implies an improvement in the use of intracellular glucose [25]. Additionally, the polyphenols of mistol fruits could affect the glucose transporters of the yeast membrane. Thereby, polyphenols could promote glucose entry into the cells. In this regard, aforegoing studies demonstrated the antihyperglycemic activity of kaempferol and procyanidin B_2_, both present in mistol PEE [8]. An increase in glucose uptake and glycogen synthesis in the cells of skeletal muscle is associated with an improvement in AMPKα activity and the expression of the Glut4 transporter, both essential for glucose entry into cells [26,27,28]. Da Costa Mousinho et al. [29] demonstrated that aqueous and methanolic extracts of *Ziziphus mucronata* cortex favored glucose consumption in C2C12 muscle cells, 3T3-L1 adipocytes, and HepG2 hepatocarcinoma cells. However, these extracts did not report a significant effect on the improvement in insulin secretion by pancreatic β cells.

### 3.2. Antiinflamatory Activity of Mistol PEE

At present, there is a wide variety of commercial anti-inflammatory drugs of different chemical natures. Non-steroidal anti-inflammatory drugs (NSAIDs), along with corticosteroids, are the most widely used for acute and chronic inflammatory pathologies, due to their high efficacy, despite the adverse effects caused by long-term use [30]. The search for natural compounds that inhibit the enzymes of the metabolic pathway of arachidonic acid, responsible for the production of pro-inflammatory mediators, is proposed as a central therapeutic objective to prevent or improve renal and cardiovascular complications in diabetes [11].

Current research reports show that low-grade inflammatory processes are associated with the risk of developing type-2 diabetes, contribute to insulin resistance, and are related to the cellular and molecular alterations that characterize metabolic syndrome, including hyperglycemia [31]. Our results showed that all extracts obtained from *Z. mistol* fruits inhibit lipoxygenase and cyclooxygenase-2 enzymes (Table 1), reaching percentages close to or higher than 90% for the seed and pulp powder extracts. The PEE activities on the lipooxygenase enzyme were higher than previously reported for *Z. mistol* whole fruit methanolic extracts and *Prosopis* species pods [32,33]. Additionally, Tran et al. [34] demonstrated the inhibitory power of triterpenoids and lignans isolated from *Z. jujuba* var. *inermis* on the expression of the COX-2 enzyme. These results could suggest that the phenolic compounds of *Z. mistol* extracts would also be inhibitors of this enzyme expression and activity.

Regarding phospholipase A_2_ (sPLA_2_), the pulp extract was the most active at inhibiting this pro-inflammatory enzyme (Table 1). The enzyme sPLA2 is the first enzyme in the arachidonic acid pathway. Thereby, the inhibition of sPLA2 activity by *Z. mistol* polyphenols may reduce the production of the arachidonic acid that would be converted to eicosanoids by COX and LOX. There are numerous reports of anti-inflammatory activity resulting from in vivo assays of other *Ziziphus* species, such as *Z. lotus* and *Z. spina-christi* [35,36]. Kadioglu et al. [36] evaluated the anti-inflammatory capacity of *Z. spina-christi* extracts, focusing on main flavonoid compounds. Through molecular docking software, the authors were able to determine the action of spinosyn, a flavonoid found in *Z. mistol* extracts [36]. Other authors reported anti-inflammatory activity for flavonoid C-glycosides derived from apigenin and kaempferol, by inhibition of COX and LOX [37,38], and anti-inflammatory activity for chalcones such as chalconaringenin and floridzine, present in polyphenol extracts obtained from the mistol. Other natural and synthetic chalcones [8,39] impacted not only on COX-2 activity but also on the iNOS enzyme. This frame shows that the bioactive compounds present in *Z. mistol* are excellent sources for the design of multi-vector drugs against inflammation, many of them with complementary activities that help reduce the different types of ailments that occur in inflammatory diseases [40,41].

### 3.3. Toxicity of Mistol PEE

The Ames test has a high predictive effect of carcinogenicity (around 80%) through diverse mechanisms like point mutations, base-pair substitutions, or frameshift mutations. For this reason, the mutagenic effect of *Z. mistol* extracts was evaluated to ensure their safe use. None of the concentrations of the PEE tested showed mutagenic effects against the TA98 or TA100 strains up to 500 µg GAE/plate, indicating the absence of mutagens in their composition that could cause base-pair substitution mutations (detected with TA100) or frameshift (recognized with TA98) (Table 2). Furthermore, all mutagenicity ratio (MR) values were lower than 2, thus indicating the absence of toxicity towards the genetic material of the strains. Similar results were previously obtained for whole-fruit extracts of *Z. mistol* [32]. Although there are no reports on the genotoxicity of *Z. mistol* seed, pulp, and skin extracts, studies indicate the absence of toxicity for *Z. jujuba* extracts on the DNA of cells such as lymphocytes, PC12 cells, and HepG2 cells [42,43,44].

Additionally, the acute toxicity of mistol PEE, using brine shrimp larvae as an organism model, was evaluated. Acute toxicity tested through the *Artemia salina* has been used for decades as a rapid and simple detection method on a laboratory scale due to its good correlation with animal models [45]. No extracts were found to be significantly cytotoxic up to concentrations of 1000 µg GAE/mL (data not shown). Similar results were found for other *Ziziphus* species, such as *Z. oxyphylla* stem extracts [46]. However, there are reports of high lethality for elevated concentrations of fruit extracts from other *Ziziphus* species using this bioassay (with lethal concentrations 50 between 8 and 45 µg/mL) [47].

As for the cytotoxicity test, none of the extracts showed cytotoxicity on human lung fibroblasts (normal cells) (GM 07492A) up to concentrations of 1000 µg GAE/mL (data not shown). Toxicity studies in cell lines allow assay sample concentrations and exposure times that can be transferred to the human condition. Although there are no reports about the cytotoxicity of mistol extracts on normal cells, there are reports of cytotoxic activity of lipids found in *Z. mistol* seeds on adenocarcinoma cells; these results were based on the study of the decrease in the incidence of murine mammary carcinoma with the application of a diet with 5% *Z. mistol* seeds oil [48].

The results obtained in toxicity evaluations on different levels, i.e., prokaryotic cells, eukaryotic cells, and complex organisms, indicate the absence of toxicity of the phenolic compounds obtained from mistol powders in the same concentration range where they have anti-inflammatory and hypoglycemic activity.

## 4. Conclusions

Although *Z. mistol* offers an interesting variety of non-wood forest products, mainly from its fruits, it is an endangered tree due to the current degraded state of the Chaco forest. Knowledge of the beneficial functional properties of mistol fruit powders can allow recovery of mistol, encouraging its sustainable consumption in Argentina as healthy food or as a complement for the prevention of metabolic disorders. The mistol powders represent a safe source of bioactive compounds for a healthy diet, diminishing the glucose bioaccessibility and modulating the inflammatory response, which makes them especially attractive for the prevention or amelioration of health conditions such as metabolic syndrome and related pathologies. However, clinical trials are essential to support these anti-inflammatory and hypoglycemic activities in humans.

## Figures and Tables

**Table 1:** Inhibitory activity of pro-inflammatory enzymes by extracts (PEE) of *Z. mistol*.

Sample	Inhibition (%) (100 μg GAE/mL)		
	sPLA2	LOX	COX-2
Seeds	NI	89.8 ± 2.3 ^a^	91.0 ± 0.9 ^c^
Pulp	46.0 ± 1.2 ^b^	83.0 ± 1.0 ^b^	96.0 ± 2.6 ^b^
Skin	25.0 ± 0.6 ^c^	65.1 ± 0.6 ^c^	68.5 ± 1.5 ^d^
Naproxen	95.00 ± 2.80 ^a^	-	-
Indomethacin	-	16.00 ± 0.80 ^d^	
Nimesulide	-	-	100.0 ± 5.0 ^a^

sPLA2: phospholipase A2; LOX: lipooxygenase; COX-2: cyclooxygenase-2. Values are expressed as percent inhibition at a fixed concentration of 100 µg GAE/mL for extract samples and 50 µg GAE/mL for naproxen, indomethacin, and nimesulide (positive controls). Results are expressed as the mean of three independent tests with SEM. NI: No inhibition shown in the concentration tested. (-): Not assayed (each standard compound was tested for a specific enzyme). Different letters in the same column show significant differences between each part of the fruit, according to Tukey’s test (*p* ≤ 0.05).

**Table 2:** Mutagenic activity of extracts (PEE) of the three parts of the *Z. mistol* fruit assayed in *Salmonella* strains.

Sample	μg GAE/Plate	N° Revertants/Plate		MR	
		TA98	TA100	TA98	TA100
Seeds	175	104 ± 17 ^b^	30 ± 7 ^b^	0.79	1.00
	250	110 ± 5 ^b^	35 ± 2 ^b^	0.83	1.16
	500	110 ± 1 ^b^	45 ± 4 ^b^	0.83	1.50
Pulp	175	100 ± 5 ^b^	16 ± 2 ^b^	0.76	0.53
	250	112 ± 12 ^b^	13 ± 1 ^b^	0.85	0.43
	500	121 ± 20 ^b^	20 ± 3 ^b^	0.92	0.64
Skin	175	99 ± 13 ^b^	19 ± 2 ^b^	0.75	0.63
	250	112 ± 5 ^b^	25 ± 5 ^b^	0.85	0.83
	500	136 ± 11 ^b^	28 ± 2 ^b^	1.03	0.93
Negative control (DMSO)		131 ± 37 ^b^	30 ± 6 ^b^		
Positive control (4-NPD)		2861 ± 114 ^a^	1998 ± 38 ^a^		

μg GAE/plate: μg of gallic acid equivalents/plate. MR: Mutagenicity Ratio. DMSO: dimethyl sulfoxide. 4-NPD: 4-nitro-*o*-phenylenediamine at a concentration of 10 μg/plate. The results correspond to mean values between triplicates of two independent tests. Different letters in the same column show significant differences between each part of the fruit, according to Tukey’s test (*p* ≤ 0.05).

## Data Availability

The datasets generated during and/or analyzed during the current study are available from the corresponding author on reasonable request.
